# Differential expression of *Nrat1* is responsible for Al-tolerance QTL on chromosome 2 in rice

**DOI:** 10.1093/jxb/eru201

**Published:** 2014-05-12

**Authors:** Jixing Xia, Naoki Yamaji, Jing Che, Ren Fang Shen, Jian Feng Ma

**Affiliations:** ^1^Institute of Plant Science and Resources, Okayama University, Chuo 2-20-1, Kurashiki, Japan; ^2^State Key Laboratory of Soil and Sustainable Agriculture, Institute of Soil Science, Chinese Academy of Sciences, Nanjing 210008, China

**Keywords:** Al QTL, Al tolerance, expression, genotypic difference, *Nrat1*, *Oryza sativa*.

## Abstract

Although rice (*Oryza sativa*) is the most Al-tolerant species among small-grain cereal crops, there is wide genotypic variation in its tolerance to Al toxicity. A number of quantitative trait loci (QTLs) for Al tolerance have been detected, but the responsible genes have not been identified. By using chromosome segment substitution lines, this work found that *Nrat1*, a gene encoding an Al transporter, is responsible for a QTL previously detected on chromosome 2. Substitution of the chromosome segment containing *Nrat1* from Koshihikari (Al-tolerant variety) by that from Kasalath (Al-sensitive variety) decreased *Nrat1* expression and Al uptake and tolerance, but increased binding of Al to the cell wall. Nrat1 in Kasalath showed tissue localization similar to Koshihikari in the roots. Although Koshihikari and Kasalath differed in four amino acids in Nrat1 protein, Nrat1 from Kasalath also showed transport activity for Al. Analysis with site-directed mutagenesis revealed that these differences did not affect the Al-transport activity much. Furthermore, there was no correlation between Al tolerance and the open-reading-frame sequence differences in other rice varieties. On the other hand, there was good correlation between *Nrat1* expression and Al tolerance; however, sequence comparison of the promoter region up to 2.1kb did not give a clear difference between the Al-tolerant and -sensitive varieties. Taken together, these results indicate that differential expression of *Nrat1* is responsible for the QTL for Al tolerance on chromosome 2, although the mechanism controlling *Nrat1* expression remains to be examined.

## Summary

Different expression of *Nrat1*, a gene encoding a plasma-membrane-localized Al transporter, is partially responsible for genotypic difference in Al tolerance in rice.

## Introduction

Aluminium (Al) toxicity is a major limiting factor of crop production on acid soils, but there is a wide difference in Al tolerance between species and cultivars within a species (Ma *et al.*, 2002; Famoso *et al.*, 2011). Among small-grain cereal crops, rice (*Oryza sativa*) shows the highest tolerance to Al toxicity, but genotypic differences also exist; usually Japonica varieties show higher Al tolerance than Indica varieties (Ma *et al.*, 2002). Furthermore, the relative degree of Al tolerance in the five subpopulations follows the order temperate japonica > tropical japonica > aromatic > indica = aus (Famoso *et al.*, 2011).

A number of quantitative trait loci (QTLs) for Al tolerance have been identified by using different mapping populations and phenotyping methods (Ma and Furukawa, 2002). Wu *et al.* (2000) identified four QTLs for Al tolerance on chromosomes 1, 3, 9, and 12, using relative root length as a physiological parameter in a random inbred mapping population derived from Azucena (Al-tolerant) and IR1552 (Al-sensitive). Five and 10 QTLs, respectively, for Al tolerance scattering on different chromosomes were detected in populations derived from Chiembau × Omon 269-65 and from CT9993 × IR62266 (Nguyen *et al.*, 2001, 2003). On the other hand, Ma *et al.* (2002) used relative root elongation as a parameter and identified three QTLs for Al tolerance on chromosomes 1, 2, and 6 in a population of 183 backcross inbred lines derived from a cross of Koshihikari (Al-tolerant) and Kasalath (Al-sensitive). Xue *et al.* (2007) mapped three QTLs for Al tolerance on chromosomes 1, 9, and 11 in a recombinant inbred line population derived from a cross between the tolerant japonica cultivar Asominori and the sensitive indica cultivar IR24 based on relative root elongation. Recently, a total of 48 distinct Al-tolerance genomic regions were detected by genome-wide association mapping based on relative root growth (Famoso *et al.*, 2011). However, the genes responsible for these QTLs have not been identified.

On the other hand, by using mutant approaches, a number of Al-tolerance genes have been identified in Japonica varieties (Ma *et al.*, 2014). *ART1* (Al-tolerance transcription factor 1), a C2H2 zinc-finger type transcription factor, was reported to be involved in Al tolerance (Yamaji *et al.*, 2009). ART1 regulates at least 31 genes by binding to the core *cis*-acting element [GGN(T/g/a/C)V(C/A/g)S(C/G)] in the promoter of these genes (Tsutsui *et al.*, 2011). The expression and localization of ART1 is not induced by Al, but the expression of downstream genes is upregulated by Al within hours. Seven ART1-regulated genes (*STAR1*, *STAR2*, *Nrat1*, *OsALS1*, *OsFRDL4*, *OsMGT1*, and *OsCDT3*) have been functionally characterized. *STAR1* and *STAR2* encode a ATP-binding domain and a membrane-binding domain, respectively, of a bacterial type ABC transporter (Huang *et al.*, 2009). The STAR1–STAR2 complex localized to vesicles transports UDP-glucose, which may be involved in cell-wall modification, resulting in decreased Al accumulation in the cell wall. OsFRDL4 is responsible for the secretion of citrate in response to Al (Yokosho *et al.*, 2011), while OsCDT3, encoding a small cysteine-rich peptide, shows binding activity with Al, thereby preventing Al entering into the root cells (Xia *et al.*, 2013). On the other hand, OsMGT1 functions as a Mg transporter (Chen *et al.*, 2012), and upregulation of *OsMGT1* could alleviate internal Al toxicity by enhancing Mg uptake. Nrat1, a member of Nramp family, takes up trivalent Al at the plasma membrane (Xia *et al.*, 2010), which is required for subsequent sequestration of Al into the vacuoles for final detoxification. Vacuolar sequestration of Al is mediated by OsALS1, a half-size ABC transporter localized to the tonoplast (Huang *et al.*, 2012). Among these genes examined, *OsFRDL4* showed a good correlation between its expression level and Al tolerance (Yokosho *et al.*, 2011), indicating that this gene may be responsible for the genotypic difference in Al tolerance. By contrast, there is no correlation between the genotypic variation of expression levels of *STAR1*, *OsMGT1*, *OsCDT3*, and *OsALS1* and Al tolerance (Huang *et al.*, 2009, 2012; Chen *et al.*, 2012; Xia *et al.*, 2013), suggesting that they are involved in fundamental Al detoxification processes common in most rice varieties.

Recently, genome-wide association detected a single, highly significant region on chromosome 2 that was unique to the aus subpopulation (Famoso *et al.*, 2011). This region, which contained the *Nrat1* candidate gene (*Os02g0131800*), is the same as the location of a QTL for Al tolerance on chromosome 2 detected previously in a population from Kasalath and Koshihikari (Ma *et al.*, 2002). This QTL explained 7.3% of the variation of Al tolerance. Physiological characterization showed that although Al-induced secretion of citrate from the roots was higher in Koshihikari than in Kasalath at higher Al concentrations, the difference between the two varieties was not significant (Ma *et al.*, 2002).

The present study investigated whether *Nrat1* is responsible for the QTL detected by using chromosomal segment substitution lines. Furthermore, expression level, tissue localization, and Al transport activity were compared between Al-tolerant and –sensitive varieties. This work found that differential *Nrat1* expression level is partially responsible for the genotypic difference in Al tolerance in rice.

## Materials and methods

### Plant materials and growth conditions

Two chromosome segment substitution lines (SL204 and SL205) were provided by the Rice Genome Resource Center (http://www.rgrc.dna.affrc.go.jp/). In SL204, the segment from marker C1357 to G132 (0–60.3 cM of chromosome 2) containing *Nrat1* was substituted by the Kasalath segment in Koshihikari background, while in SL205, the segment from marker G132 to C747 (60.3–107.7 cM of chromosome 2) was substituted (Supplementary Fig. S1 available at *JXB* online), which was used as a negative control. Rice seeds (Kasalath, Koshihikari, SL204, SL205) were soaked in tap water overnight at 30 °C in the dark and then transferred to a net floating on 0.5mM CaCl_2_ in a 1.5-l plastic container. Seedlings were grown for 4–7 d at 25 °C. Similar size seedlings were selected and used for the following experiments.

### Evaluation of Al tolerance

Six rice seedlings (5-d-old) per each genotype were exposed to 0.5mM CaCl_2_ containing 0, 30, or 50 μM Al (pH 4.5) for 24h. Root lengths were measured with a ruler before and after treatments. Relative root elongation was calculated as follows: (root elongation with Al) / (root elongation without Al) × 100. Six roots were measured for each treatment.

### Al determination in cell sap and cell wall

For determining Al accumulation in the root tips, 5-d-old seedlings (Kasalath, Koshihikari, SL204, SL205) were exposed to 50 μM Al (pH 4.5) for 8h and then root segments (0–1cm, 20 roots each) were excised after washing three times with 0.5mM CaCl_2_. To obtain root cell sap, the root segments were put in ultra-free-MC centrifugal filter units (Millipore) and centrifuged at 3000 *g* for 10min at 4 °C to remove apoplastic solution. The roots were then frozen at –80 °C overnight. The root cell sap solution was obtained by thawing the samples at room temperature and centrifuging at 20 400 *g* for 10min. The residual cell wall were washed three times with 70% ethanol and then immersed in a 0.5ml of 2M HCl for at least 24h with occasional vortex. The Al concentration in solution was determined by inductively coupled plasma mass spectrometry (ICP-MS, 7700X, Agilent Technologies).

### RNA isolation and gene expression analysis

To examine the expression pattern of *Nrat1*, rice seedlings (7-d-old) were exposed to different Al concentrations (0–50 μM) for 8h. Root tips (0–1cm) were excised. All samples with three replicates were subjected to RNA extraction. Total RNA was extracted using an RNeasy Mini Kit (Qiagen). Total RNA (1 μg) was used for first-strand cDNA synthesis using a SuperScript II kit (Invitrogen), following the manufacturer’s instructions, with an oligo(dT)_12–18_ primer. Expression was determined with SYBR Premix Ex Taq (Takara) by Mastercycler ep realplex (Eppendorf). The primer sequences used for reverse-transcription PCR were as follows: *Nrat1*, forward 5′-GAG GCCGTC TGCAGGAGAGG-3′ and reverse 5′-GGAAGT ATCTGCAAGCAGCTCTGATGC-3′; and *ART1*, forward 5′-CAGTGCTTCT CGTGGGTCTT-3′ and reverse 5′-CCTG TGCGTGAA GAACCACT-3′. *HistoneH3* (forward 5′-AGTTT GGTCGCTCTCGATTTCG-3′; reverse 5′-TCAAC AAGTTGACCACGTCACG-3′) was used as an internal control.

### Immunohistological staining

Immunostaining with an antibody specific to Nrat1 was performed, obtained by immunizing rabbits with the synthetic peptide N-MEGTGEMREVGRETLHGG-C (positions 1–18 of Nrat1) (Xia *et al.*, 2010). After the roots of Koshihikari and Kasalath were exposed or not to 30 μM Al for 12h, the root tips (2mm) were excised. The procedures for immunostaining were the same as described previously (Yamaji and Ma, 2007).

### Yeast experiments

The cDNA fragment containing the entire open reading frame (ORF) for *Nrat1* derived from Koshihikari or Kasalath was amplified by PCR using the primers 5′-GGTAC CAAAAT GGAAGGG ACTGGTGAGATGA-3′ and 5′-CTACATGGAAGCATCGGCA A-3′ (forward and reverse, respectively). The fragment was first cloned into the pGEM-T vector (Promega). After sequence confirmation, *Nrat1* cDNA was excised with *Kpn*I and *Not*I for cloning into pYES2 (Invitrogen). Site-directed mutants of *Nrat1* (Koshihikari) were generated on the Nrat1-pYES2 construct by PCR using the following synthetic oligonucleotide primers: common primers as for amplification; for mutation of *Nrat1* (Koshihikari): E120K, forward 5′-GAGTACCCGCACTATGTCAACA-3′ and reverse 5′-TGTTG ACATAGTGCGGGTACTC-3′; V326I, forward 5′-GATCT CCCCA AAATATTCCTGAGTAAAAGAGGTGTGG-3 and reverse 5′-AC TCAG GAATAT TTTGGGGAGATCAAGCTCGGTG-3′; T500M, forward 5′-CAGGAA GGACATGGTGGCGACGTACGTGC CG-3′ and reverse 5′-ACGTCG CCAC CATG TCCTTCCTGA ACGTCAGGT-3′; and V515A, forward 5′-GTGG CGACG TACGT GCCGGTGC-3′ and reverse 5′-GCACCGGCACGTACGTCGC CAC-3′. Using combinations of these primers for PCR, four mutant types of *Nrat1* (Koshihikari) were generated. Mutation in each amplified fragment was confirmed by DNA sequencing. These fragments were then cloned into pYES2 (Invitrogen). The resulting plasmids were introduced into *Saccharomyces cerevisiae* BY4741.

Al sensitivity evaluation on agar and Al uptake in liquid culture were performed as described by Xia *et al.* (2010). For Al sensitivity evaluation, *Nrat1* (Koshihikari), *Nrat1* (Kasalath), and vector control pYES2 were transformed into *S. cerevisiae* BY4741 and then grown on solid media (LPM without uracil; MacDiarmid and Gardner, 1996) containing 0, 200, or 300 μM Al buffered with 5mM succinic acid. For Al uptake in liquid culture, transformants were selected on LPM without uracil and grown in synthetic complete medium without uracil. Cells at midexponential phase were harvested and transferred to LPM without uracil. Cells were cultured for 2h, followed by addition of AlCl_3_ to the medium to a final concentration of 50 μM Al. After 4h of incubation with shaking, cells were harvested by centrifugation. The pellets were washed three times with milli-Q ultrapure water (Millipore) and digested with 2M HCl. The concentration of Al in the digest solution was determined by ICP-MS.

### Western blot analysis of Nrat1

Total protein (5 μg) extracted from the yeast expressing different alleles of *Nrat1* were mixed with sample buffer containing 100mM Tris-HCl (pH 6.8), 4% (w/v) SDS, 20% (w/v) glycerol, 0.01% (w/v) bromophenol blue, and 200mM β-mercaptoethanol. The mixture was allowed to incubate at 65 °C for 10min and SDS-PAGE was run using 5–20% gradient polyacrylamide gels (ATTO, Tokyo, Japan). After electrophoresis, the gel was transferred to polyvinylidene difluoride membrane with a semidry blotting system for Western blot analysis. The membrane was treated with the primary antibody anti-Nrat1 or anti-H^+^ATPase (a plasma membrane protein as an internal standard) diluted at 1:500 for 1h. Then the membrane was washed and incubated with anti-rabbit IgG HRP conjugate (1:10 000 dilution, Promega) for 1h at room temperature. The ECL Plus Western Blotting Detection System (GE Healthcare) was used for detection of signals via chemiluminescence.

### Sequencing of promoter and open reading frame of *Nrat1*

To compare the promoter sequences, nine rice cultivars were used: Kasalath and three cultivars (Miryang-23, PI312777, IR36) of the Indica subspecies and five cultivars (Koshihikari, Nipponbare, Nagoyashiro, Mack Kheua and Mu Bang) of the Japonic subspecies. The 2.1-kb region upstream of the initiation codon of *Nrat1* was amplified by PCR from their genomic DNA using primers 5′-GGTACCAACACGTCTGACGCTTGTT-3′ and 5′-CTCGAG ATTCTATGTTGCTAATGCACCTTGT-3′ (forward and reverse, respectively). To clone the full-length ORF sequence of *Nrat1*, total RNA was extracted from rice roots using a RNeasy Plant Mini Kit and then converted to cDNA using the protocol supplied by the manufacturers of SuperScript II. The full-length ORF was amplified by PCR using the primers used for amplification of *Nrat1*. The fragments of the amplified promoters and cDNAs were cloned into the vector pGEM-T (Promega) and the sequences were confirmed using a sequence analyser (ABI PRISM 310 Genetic Analyzer, Applied Biosciences).

### Genotypic difference in *Nrat1* expression and Al tolerance

To determine the expression level of *Nrat1* among nine rice cultivars as described above, 5-d-old seedlings were exposed to a 0.5mM CaCl_2_ containing 50 μM Al for 8h. After treatment, the roots from each cultivar were sampled for RNA extraction and used for quantitative analysis of gene expression, as described above.

To compare Al tolerance, 5-d-old seedlings of nine rice cultivars, as described above, were exposed to 0.5mM CaCl_2_ containing 0 or 50 μM Al for 24h. The root length was measured with a ruler before and after treatment. Ten roots were measured for each treatment.

## Results

### *Nrat1* is responsible for Al QTL on chromosome 2

**Fig. 1.**
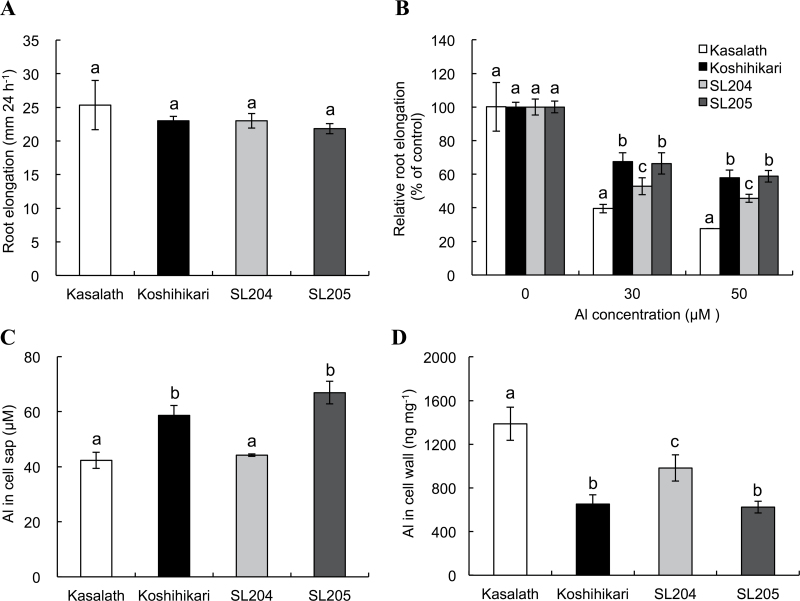
Physiological characterization of two chromosome segment substitution lines. (A, B) Root elongation in 5-day-old seedlings of Koshihikari, Kasalath, SL204, and SL205 over 24h; without Al (A; 0.5mM CaCl_2_, pH 4.5) and with Al (B; 0.5mM CaCl_2_, pH 4.5, with 0, 30, or 50 μM Al); root length was measured before and after treatment and elongation relative to root elongation without Al was calculated; data are mean±SD of six biological replicates. (C, D) Al concentration in cell sap (C) and cell walls (D) of root tips; roots of Koshihikari, Kasalath, SL204, and SL205 were exposed to 0.5mM CaCl_2_ (pH 4.5) containing 50 μM Al for 8h; root tips (0–1cm) were excised and Al concentration was determined by ICP-MS; data are mean±SD of three biological replicates. Different letters above the bars indicate significant differences (*P*<0.05, Tukey’s test).

Three QTLs controlling Al tolerance were previously detected on chromosomes 1, 2, and 6 by using 183 backcross inbred lines derived from Koshihikari (Al tolerant) and Kasalath (Al sensitive) (Ma *et al.*, 2002). A recently identified Al-tolerance gene, *Nrat1*, is located to the QTL region on chromosome 2. To examine whether *Nrat1* is responsible for the QTL, this work obtained two chromosome segment substitution lines, which contained the *Nrat1* allele from Kasalath (SL204) or not (SL205 as a negative control) in Koshihikari background (Supplementary Fig. S1 available at *JXB* online). First, Al tolerance to different Al concentrations was compared among Koshihikari, Kasalath, SL204, and SL205. In the absence of Al, the four lines showed similar root elongation (Fig. 1A). However, in the presence of 30 and 50 μM Al, relative root elongation was significantly lower in Kasalath and SL204 than in Koshihikari and SL205 (Fig. 1B). Relative root elongation of SL204 was higher than that of Kasalath, indicating that other QTLs also contribute to Al tolerance in Koshihikari. This is consistent with this study group’s previous results (Ma *et al.*, 2002).

Nrat1 is an Al transporter localized to the plasma membrane of the root cells and knockout of *Nrat1* results in decreased Al uptake (Xia *et al.*, 2010). If *Nrat1* is responsible for the QTL on chromosome 2, SL204 should show a phenotype similar to the knockout line. Therefore, Al concentrations in the root cell sap were compared. At the root tip (0–1cm), Al concentration in root cell sap was significantly lower in Kasalath and SL204 than in Koshihikari and SL205 (Fig. 1C) and Al content in cell walls was higher in Kasalath and SL204 than in Koshihikari and SL205 (Fig. 1D).

**Fig. 2.**
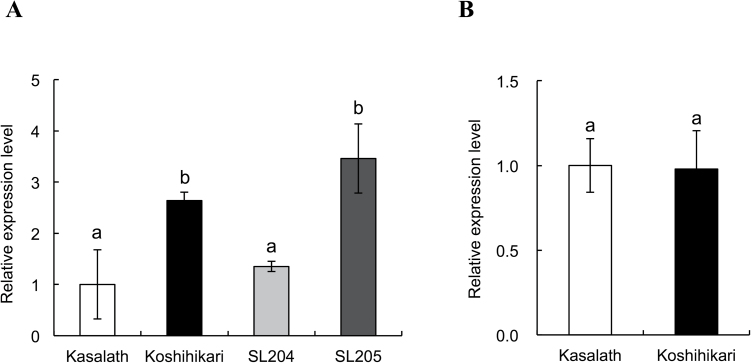
Expression of *Nrat1* (A) and *ART1* (B) in roots of two chromosome segment substitution lines; 7-d-old rice seedlings were exposed to 50 μM Al for 8h; RNA was extracted from root tips (0–1cm) and *HistoneH3* was used as an internal standard. Data are mean±SD of three biological replicates. Different letters above the bars indicate significant differences (*P*<0.05, Tukey’s test).

Furthermore, this work compared the expression level of *Nrat1* in the root tips of Koshihikari, Kasalath, SL204, and SL205. In the absence of Al, the expression level of *Nrat1* was almost double in Koshihikari than in Kasalath at root tips (Supplementary Fig. S2 available at *JXB* online). In the presence of Al, *Nrat1* was induced in both Kasalath and Koshihikari, but Kasalath showed expression of *Nrat1* lower than Koshihikari (Fig. 2A). Substitution of the *Nrat1* allele from Koshihikari with that from Kasalath resulted in decreased expression of *Nrat1* to a similar level of Kasalath (SL204, Fig. 2A), whereas substitution of other regions did not affect the expression of *Nrat1* (SL205, Fig. 2A).

### Expression analysis of *ART1*

A previous study has shown that the expression level of *Nrat1* is regulated by *ART1* (Xia *et al.*, 2010). To investigate whether differential expression of *Nrat1* is caused by variations in *ART1* expression between Kasalath and Koshihikari, this work compared the expression of *ART1* in two varieties. There was no difference in the expression level of *ART1* in the root tips between Kasalath and Koshihikari (Fig. 2B).

### Comparison of the promoter and coding region sequences of *Nrat1* between Koshihikari and Kasalath

To understand the mechanisms underlying different expression of *Nrat1* in two varieties, this work compared the promoter sequence of *Nrat1* up to 2.1kb upstream from the translation initiation site. Five nucleotide substitutions and a 5-bp insertion were found in the promoter region of *Nrat1* of Kasalath (Supplementary Fig. S3 available at *JXB* online). However, the number of *cis*-acting elements of *ART1* was the same in the promoters between two varieties (Supplementary Fig. S3 available at *JXB* online).

This work also compared the sequence of the *Nrat1* coding region between Koshihikari and Kasalath. There were five nucleotide differences, resulting in four amino acid changes at positions E120K, V326I, T500M, and V515A (Supplementary Fig. S4 available at *JXB* online). Predication with SOSUI showed that these amino acid changes did not affect the topology of Nrat1, which contains 10 transmembrane domains (Supplementary Fig. S5 available at *JXB* online).

### Localization of Nrat1 protein

**Fig. 3.**
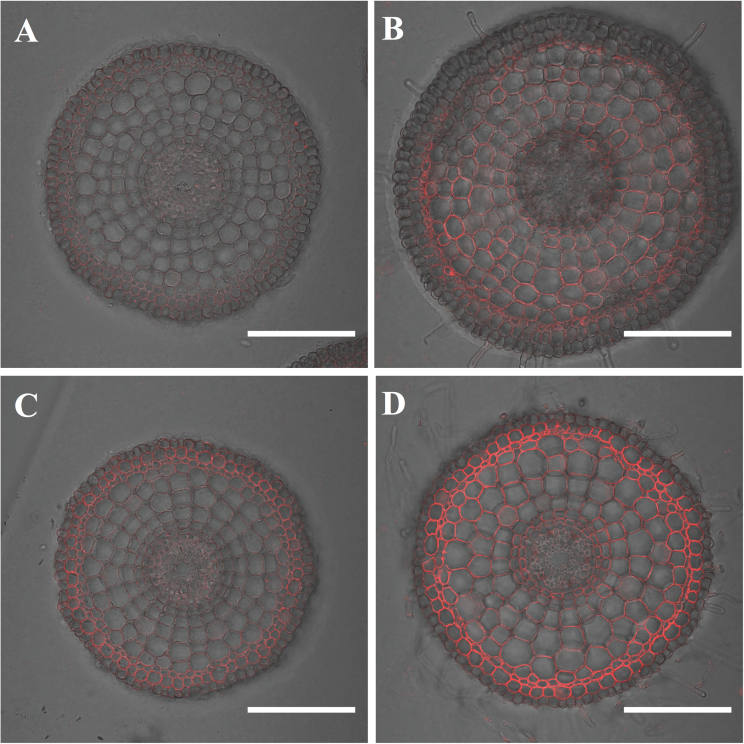
Localization of Nrat1 in roots. Immunostaining with an anti-Nrat1 antibody was performed in 2mm from the root apex of Kasalath (A, B) and Koshihikari (C, D) in roots exposed for 12h to 0.5mM CaCl_2_ without Al (A, C) or 0.5mM CaCl_2_ containing 30 μM Al (B, D). Bars, 100 μm.

Nrat1 has been reported to be localized to all cells of root tips except epidermal cells in a Japonica variety, Nipponbare (Xia *et al.*, 2010). To examine whether the differences in the promoter and coding region of *Nrat1* affected localization in tissues, this work performed immunostaining using an antibody specifically against Nrat1. In the root tip (2mm from the root tip), Nrat1 was localized to all root cells except the epidermal cells in both Koshihikari and Kasalath (Fig. 3). Furthermore, the signal was enhanced in the roots exposed to Al in both varieties. Compared with Kasalath, the signal in the Al-exposed roots of Koshihikari was stronger (Fig. 3).

### Transport activity of Nrat1 for Al in yeast

**Fig. 4.**
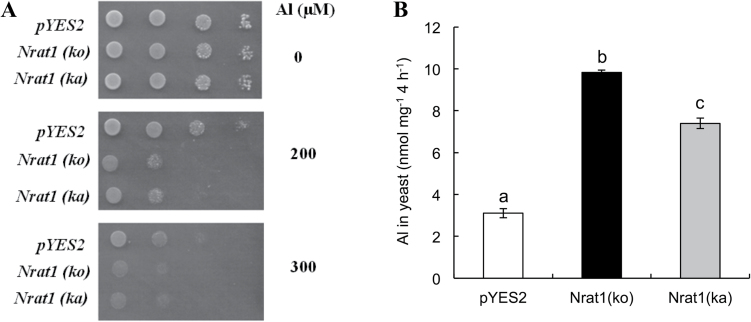
Transport activity of Nrat1 for aluminium in yeast. (A) Effect of *Nrat1* expression on Al tolerance; *S. cerevisiae* BY4741 carrying empty vector pYES2, *Nrat1* (Kasalath), or *Nrat1* (Koshihikari) were spotted on LPM without uracil medium (from left to right: 10 μl cell suspension with OD 0.2, 0.02, 0.002, and 0.0002) with or without AlCl_3_ at different dilutions and incubated at 30 °C for 3 d. (B) Transport activity of Nrat1 (Kasalath) and Nrat1 (Koshihikari) for Al^3+^; yeast cells expressing different *Nrat1* alleles were exposed for 4h to 50 μM AlCl_3_ (pH 4.2); Al in the yeast was determined by ICP-MS after digestion with 2M HCl; data are mean±SD of three biological replicates. Different letters above the bars indicate significant differences (*P*<0.05, Tukey’s test).

To test whether amino acid substitutions in the Nrat1 protein affect the transport activity for Al, this work expressed different *Nrat1* alleles in yeast and compared Al uptake. Expression of *Nrat1* alleles from both Kasalath and Koshihikari did not affect yeast growth in the absence of Al (Fig. 4A). However, in the presence of Al, expression of *Nrat1* from either Koshihikari or Kasalath increased the sensitivity of yeast to Al toxicity (Fig. 4A), indicating that Nrat1 from Kasalath also has transport activity for Al.

To quantify Al uptake, Al content in yeast expressing different *Nrat1* alleles was determined. The Al uptake ability of Nrat1 from Koshihikari was 34% higher than that from Kasalath (Fig. 4B). Since this difference might result from different expression levels of Nrat1 proteins in yeast, protein levels were determined by Western blot with an antibody specific to Nrat1. The results showed that yeast expressing *Nrat1* from Koshihikari and Kasalath produced similar levels of Nrat1 (Supplementary Fig. S6 available at *JXB* online).

**Fig. 5.**
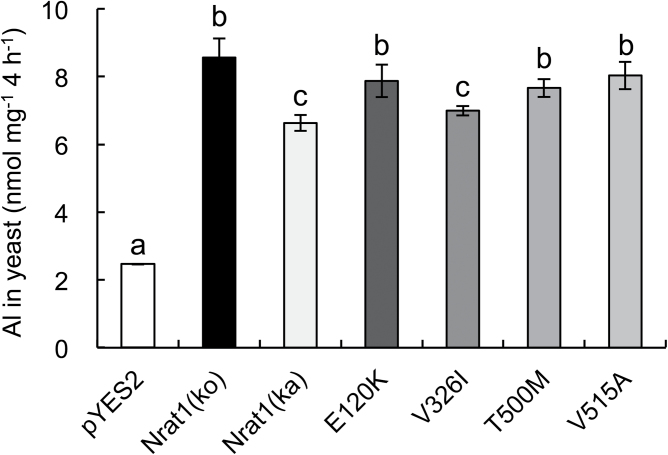
Site-directed mutagenesis analysis. Yeast cells expressing *Nrat1* (Kasalath), *Nrat1* (Koshihikari), or mutated *Nrat1* (E120K, V326I, T500M, or V515A from Koshihikari) were exposed for 4h to 50 μM AlCl_3_ (pH 4.2). Al concentration in cells was determined by ICP-MS after digestion with 2M HCl. Data are mean±SD of three biological replicates. Different letters above the bars indicate significant differences (*P*<0.05, Tukey’s test).

Since Nrat1 proteins from Koshihikari and Kasalath differ in four amino acids, this work performed a site-directed mutation analysis to examine which amino acid is important for Al transport activity. It was found that a mutation at the position of 326 from V to I seemed to slightly reduce Al transport activity (Fig. 5), whereas other mutations (E120K, T500M, and V515A) did not affect Al transport activity.

### Genotypic differences in *Nrat1* expression and promoter and ORF sequences

**Fig. 6.**
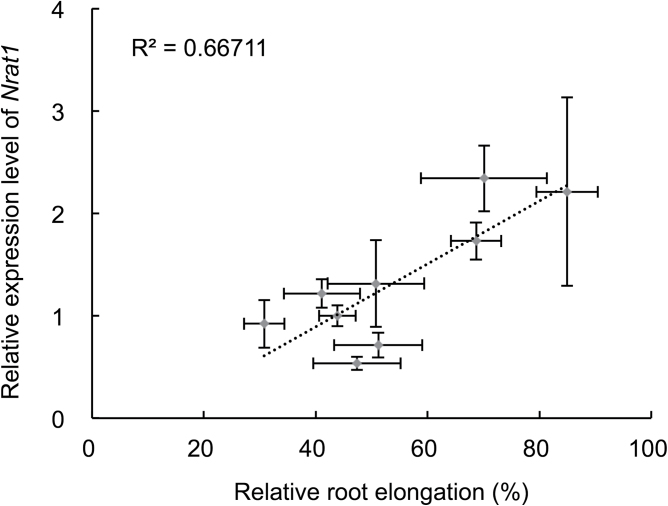
Correlation between *Nrat1* expression and Al tolerance in nine varieties. Seedlings of nine rice cultivars were exposed for 8h to 50 μM Al. Expression of *Nrat1* in the roots was determined by quantitative real-time PCR. *HistoneH3* was used as an internal standard; expression relative to Kasalath is shown; data are mean±SD of three biological replicates. Al tolerance was evaluated by exposing the seedlings to 0 or 50 μM Al for 24h and root length was measured with a ruler before and after treatment; data are mean±SD of 10 roots.

To further investigate whether *Nrat1* is responsible for genotypic difference in Al tolerance in other varieties, the expression levels and Al tolerance were compared in nine varieties differing in Al tolerance. A good correlation (*r*=0.82) was found between *Nrat1* expression level and Al tolerance (relative root elongation), irrespective of the subspecies (Fig. 6).

This work also compared the promoter (2.1kb) sequence in nine varieties. However, there was no consistent trend between promoter sequence and Nrat1 expression (Supplementary Fig. S3 available at *JXB* online). Also, no trend was found between amino acid sequence and Al tolerance in nine varieties (Supplementary Fig. S4 available at *JXB* online).

## Discussion

Nrat1 belongs to the Nramp (natural resistance-associated macrophage protein) family but, unlike other members, it transports trivalent Al (Xia *et al.*, 2010). Knockout of *Nrat1* results in decreased Al tolerance of rice (cv. Nipponbare, a *Japonica* variety) and decreased Al uptake (root cell sap concentration) but increased Al binding to the cell wall (Xia *et al.*, 2010). Therefore, Nrat1 is involved in Al tolerance in rice by transporting Al into the root cells prior to final detoxification in the vacuoles, which is mediated by a tonoplast-localized transporter, OsALS1 (Xia *et al.*, 2010; Huang *et al.*, 2012).

The location of *Nrat1* is within the region of a QTL for Al tolerance on chromosome 2 (Ma *et al.*, 2002; Yamaji *et al.*, 2009). Furthermore, another recent study with genome-wide association also showed that variations in *Nrat1* are probably responsible for the genotypic variation in Al tolerance in the aus subpopulation (Famoso *et al.*, 2011). The present study confirmed this possibility by using chromosome segment substitution lines. When the *Nrat1* region of Koshihikari (Al-tolerant) was substituted by that of Kasalath (Al-sensitive), the expression of *Nrat1* was reduced (Fig. 2A) and Al tolerance was also decreased (Fig. 1B). Furthermore, the Al concentration in the root cell sap was decreased (Fig. 1C) but that in the cell wall was increased (Fig. 1D). These phenotypes are similar to a *Nrat1* mutant, although the extent of these phenotypic changes is different (Xia *et al.*, 2010). Furthermore, there was a good correlation between *Nrat1* expression and Al tolerance in other varieties differing in Al tolerance (Fig. 6). Taken together, these results support that *Nrat1* is responsible for the QTL detected on chromosome 2. In addition, SL204 was more tolerant to Al compared with Kasalath (Fig. 1B), suggesting that other QTLs controlling Al tolerance exist in Koshihikari. This is in agreement with previous results that have shown that there are two QTLs for Al tolerance on chromosomes 1 and 2 detected in Koshihikari (Ma *et al.*, 2002).

Variations in *Nrat1* may result from protein mutation and different gene expression. The localization of Nrat1 protein to the root was similar in Koshihikari and Kasalath (Fig. 3), but there are four amino acid differences in Nrat1 between Koshihikari and Kasalath, at positions E120K, V326I, T500M, and V515A (Supplementary Fig. S4 available at *JXB* online). These changes do not seem to affect the membrane topology of Nrat1 (Supplementary Fig. S5 available at *JXB* online). When the *Nrat1* allele from Kasalath was expressed in yeast, it also showed transport activity for Al (Fig. 4B), indicating that the loss of function does not result from variation in Nrat1. Quantitative analysis showed that Al uptake mediated by the *Nrat1* allele from Koshihikari was slightly higher than that from Kasalath (Fig. 4B). Furthermore, an amino acid change at the position of 326 from V to I seems important to Al transport activity (Fig. 5). Genome-wide association analysis (Famoso *et al.*, 2011) suggested that an amino acid change from valine to alanine (but should be threonine to methionine, amino acid 500 in the paper) is responsible for natural variation in Nrat1 because this amino acid site was predicted to be involved in PKA-type AGC kinase phosphorylation. However, site-directed mutation analysis showed that this mutation did not affect the transport activity for Al in yeast (Fig. 5). Overall, alterations in protein sequence are unlikely to be a major factor responsible for genotypic difference in Al tolerance because these alterations were not found in other Al-sensitive varieties (Supplementary Fig. S4 available at *JXB* online). However, the possibility could not be excluded that all four mutations are required for a difference in Al transport activity, which remain to be examined in future.

By contrast, the expression level of *Nrat1* is responsible for the genotypic differences in Al tolerance. This is supported by a good correlation between *Nrat1* expression level and Al tolerance in different rice varieties (Fig. 6). The expression of *Nrat1* is regulated by ART1 (Yamaji *et al.*, 2009; Xia *et al.*, 2010), therefore, different expression of *Nrat1* may be caused by variation of ART1. However, there was no difference in *ART1* expression between Koshihikari and Kasalath (Fig. 2B). The numbers of *cis*-acting elements recognized by ART1 in the *Nrat1* promoter region were also similar between two varieties (Supplementary Fig. S3 available at *JXB* online). These results indicate that ART1 is unlikely to be a factor responsible for the genotypic difference in *Nrat1* expression.

Alterations in the promoter region have been revealed to be the mechanisms for regulating the expression of Al-tolerance genes in several studies (Delhaize *et al.*, 2012; Ma *et al.*, 2014). For example, the high expression level of *ALMT1* is associated with tandem repeated elements in the promoter region in most Al-tolerant lines of wheat (Sasaki *et al.*, 2006; Ryan *et al.*, 2010). In sorghum, tourist-like miniature inverted-repeat transposable elements in the promoter region of *SbMATE* were suggested to be involved in regulating the expression of this gene (Magalhaes *et al.*, 2007). In barley, a 1-kb insertion (CACTA-like transposon) in the 5′-untranscribed region of *HvAACT1* enhances its expression in Al-tolerant accessions (Fujii *et al.*, 2012). Recently, the higher expression level of *TaMATE1B* in several Brazilian wheat lines was found to be associated with the presence of a Sukkula-like transposable element (11.1-kb) in its promoter (Tovkach *et al.*, 2013). More recently, in Yorkshire fog (*Holcus lanatus*), higher *HlALMT1* expression was achieved by an increase in the number of *cis*-acting elements for transcription factor *HlART1* in the promoter region (Chen *et al.*, 2013). Maron *et al.* (2013) found that Al-tolerant cultivars of maize have three copies of *ZmMATE1* in the genome, which are identical and part of a tandem triplication. Therefore, this work compared the promoter sequences of *Nrat1* in Al-sensitive and Al-tolerant rice varieties. However, no clear differences were found in the promoter region up to 2.1kb (Supplementary Fig. S3 available at *JXB* online). There are two possibilities for this result: either the regulatory factor is present further upstream of the promoter region or the expression of *Nrat1* may be regulated differently. Further work is required to understand the mechanism underlying the *Nrat1* expression.

In conclusion, these results show that differential expression of *Nrat1* is mainly responsible for the Al-tolerance QTL located on chromosome 2 in rice. Enhancement of *Nrat1* expression in Al-sensitive varieties may increase their Al tolerance.

## Supplementary material

Supplementary data are available at *JXB* online.

Supplementary Fig. S1. Genotypes of SL204 and SL205.

Supplementary Fig. S2. Expression level of *Nrat1* in two rice cultivars with and without Al exposure.

Supplementary Fig. S3. Alignment of 2.1-kb promoter region of *Nrat1* from Kasalath, Koshihikari, and other varieties.

Supplementary Fig. S4. Alignment of the amino acid sequence of Nrat1 from Kasalath, Koshihikari, and other varieties.

Supplementary Fig. S5. Transmembrane domains of Nrat1 from Kasalath and Koshihikari predicated by SOSUI.

Supplementary Fig. S6. Western blot analysis for Nrat1 expressed in yeast.

Supplementary Data
