# Supplementary material for: Differential expression of Nrat1 is responsible for Al-tolerance QTL on chromosome 2 in rice
Source: J Exp Bot. 2014 May 12;65(15):4297–304. doi: 10.1093/jxb/eru201 (PMC4112633; doi:10.1093/jxb/eru201)
Supplement: Supplementary Data [file supp_65_15_4297__index.html]

Differential expression of Nrat1 is responsible for Al-tolerance QTL on chromosome 2 in rice — Differential expression of Nrat1 is responsible for Al-tolerance QTL on chromosome 2 in rice — Supplementary Data 

# Differential expression of *Nrat1* is responsible for Al-tolerance QTL on chromosome 2 in rice

## Supplementary Data

Data files

**Files in this Data Supplement:**

- Supplementary Data - Supplementary Data
