# Supplementary material for: Differential expression of Nrat1 is responsible for Al-tolerance QTL on chromosome 2 in rice
Source: J Exp Bot. 2014 May 12;65(15):4297–304. doi: 10.1093/jxb/eru201 (PMC4112633; doi:10.1093/jxb/eru201)
Supplement: Supplementary Data [file supp_eru201_jexbot119941_file001.pdf]

Differential expression of *Nrat1* gene is responsible for Al tolerance QTL on chromosome 2 in rice

Authors
Jixing Xia, Naoki Yamaji, Jing Che, Ren Fang Shen and Jian Feng Ma

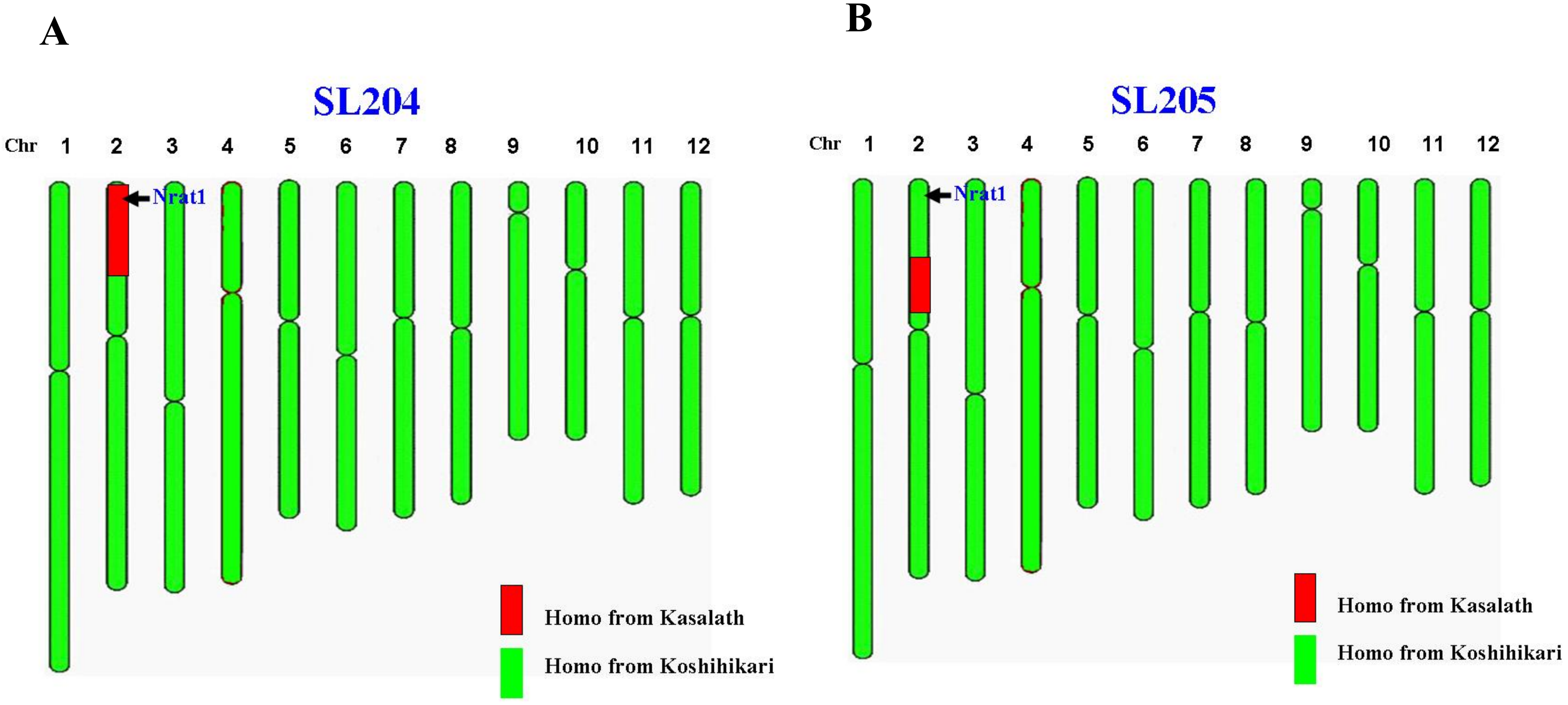

Fig. S1. Genotypes of SL204 (A) and SL205 (B). Red portions refer to the segment derived from Kasalath. Green portions refer to the segment derived from Koshihikari. Arrow indicates the location of Nrat1.

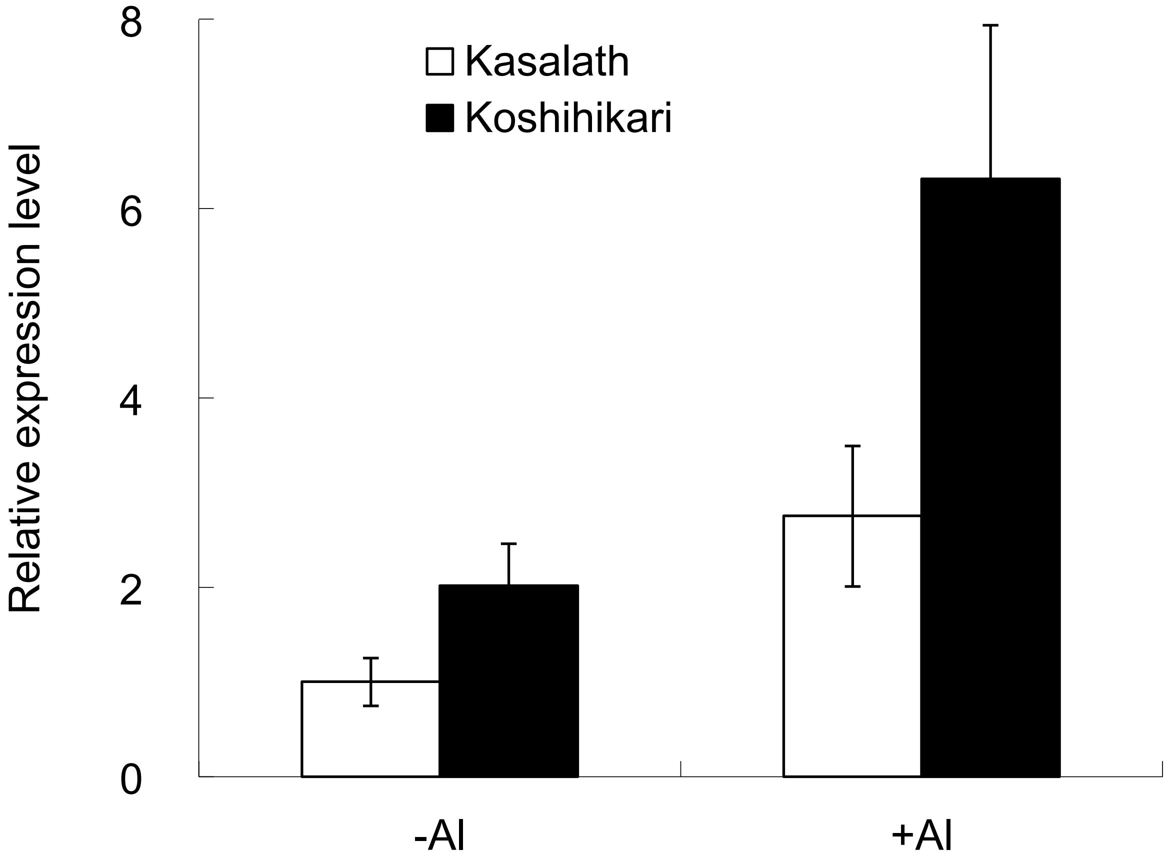

Fig. S2. Expression level of *Nrat1* in two rice cultivars. Root tips (0-1 cm) were taken from seven-day-old seedlings of Kasalath and Koshihikari exposed to 0 and 50 μM Al and subjected to RNA extraction. *HistoneH3* was used as an internal standard. Data relative to Kasalath are shown. Data are means±SD of three biological replicates.

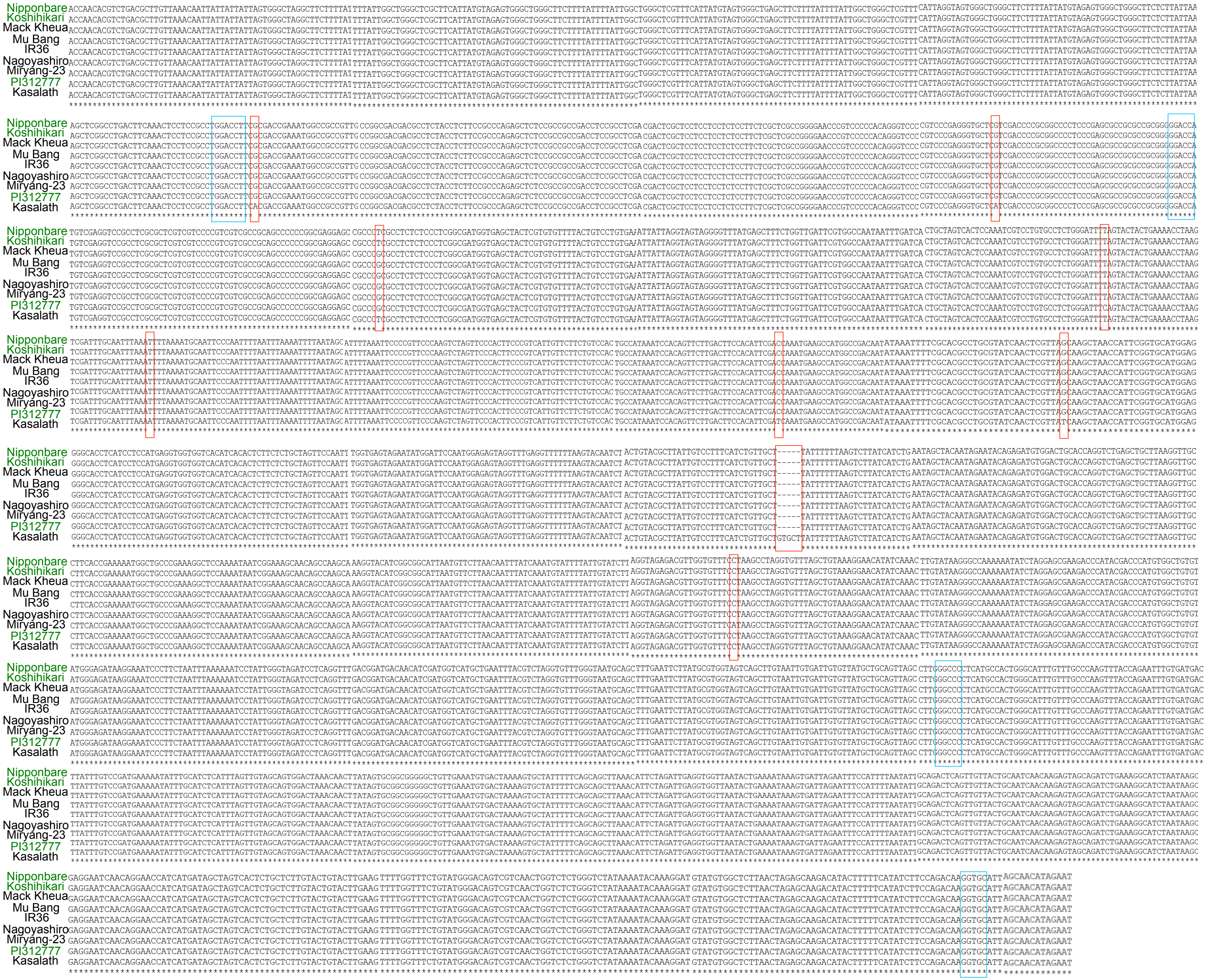

Fig. S3. Alignment of 2.1-kb promoter region of *Nrat1* from Kasalath, Koshihikari and other varieties. Different sites are boxed with red color. The cis-acting element for ART1 is boxed with blue color. Al-sensitive and –tolerant varieties were shaded with black and green colors, respectively.

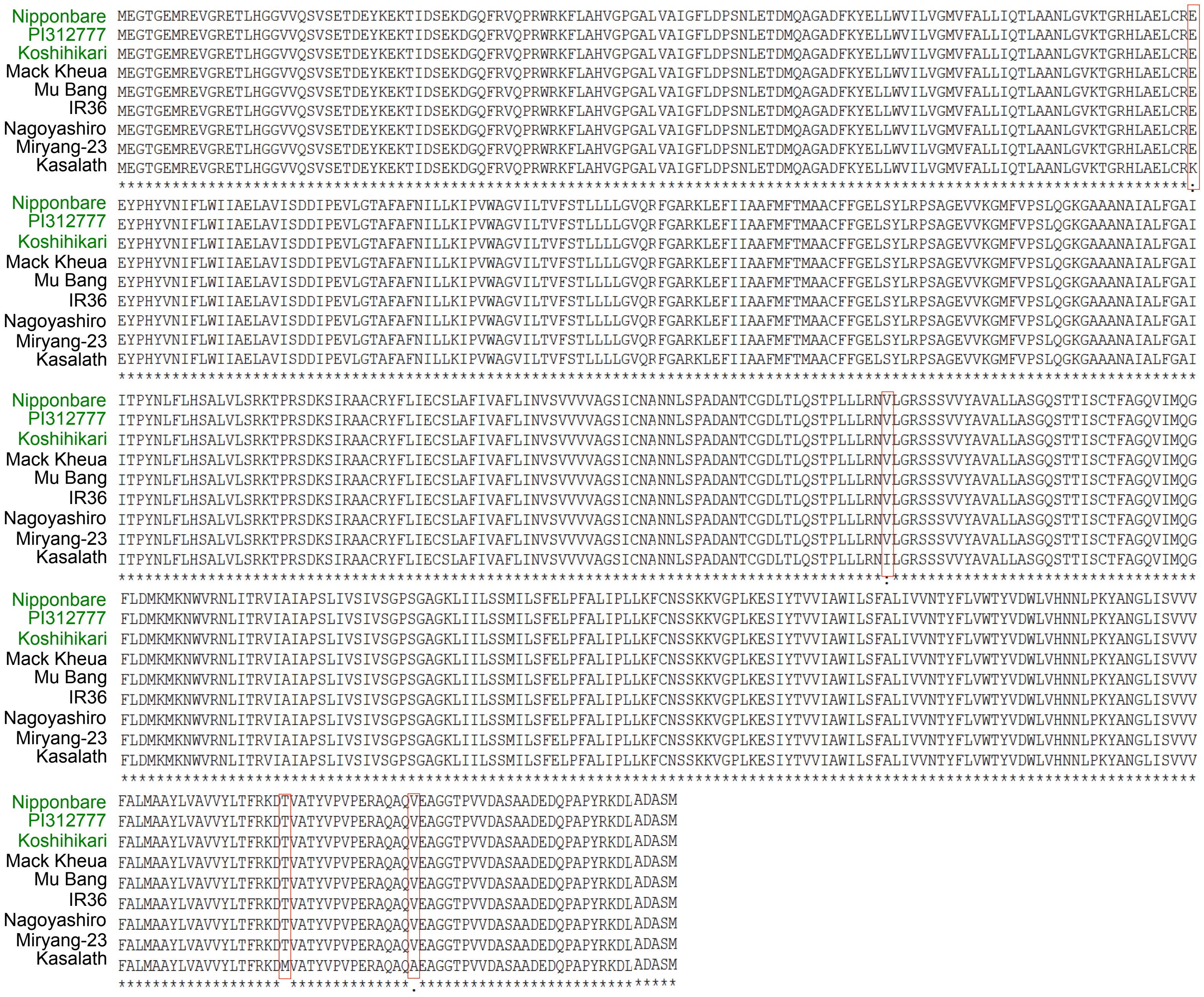

Fig. S4. Alignment of the amino acid sequence of Nrat1 from Kasalath, Koshihikari, and other varieties. Different sites are boxed with red color. Al-sensitive and -tolerant varieties were shaded with black and green colors, respectively.

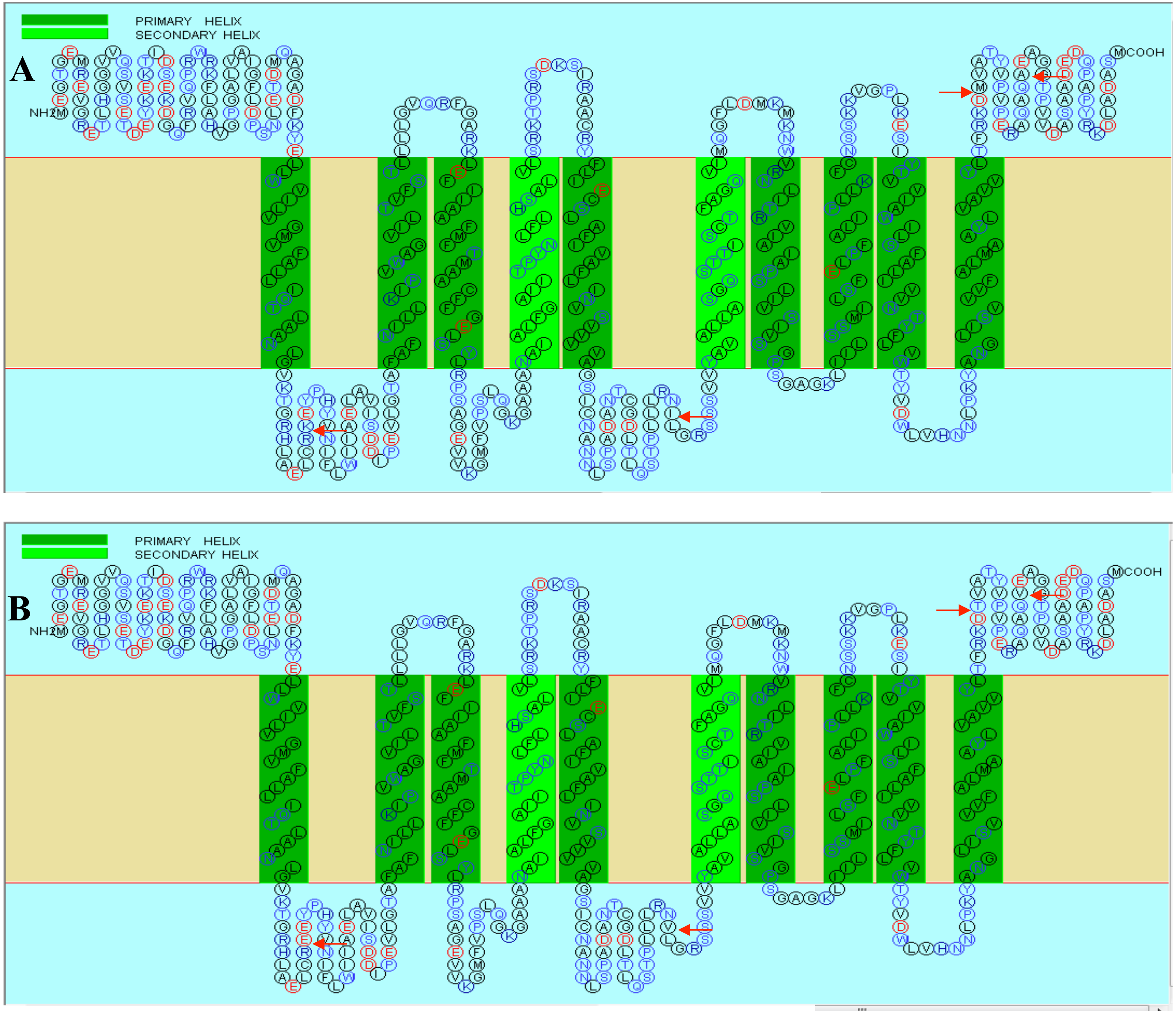

Fig. S5. Transmembrane domains of Nrat1 from Kasalath and Koshihikari predicated by SOSUI program. (A) Nrat1 from Kasalath. (B) Nrat1 from Koshihikari. Red arrows indicate different sites in proteins.

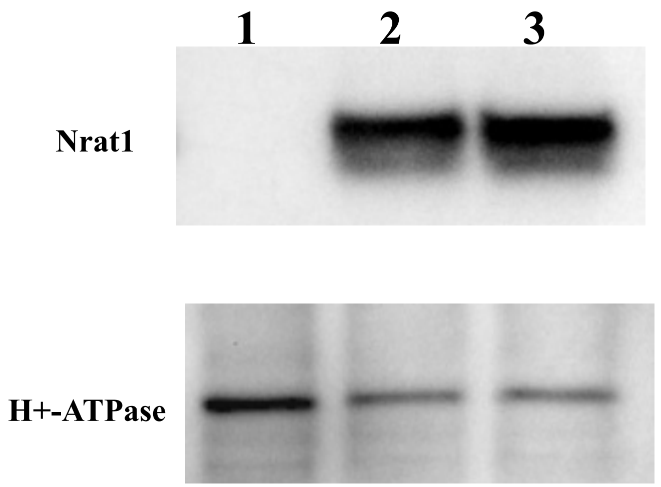

Fig. S6. Western blot analysis for Nrat1 expressed in yeast. Western blotting using antibody specific for Nrat1 or H+-ATPase. Lane 1, vector control; Lane 2-3, total protein extracted from yeast cell carrying Nrat1 (Koshihikari) and Nrat1 (Kasalath), respectively.
